# Medial Efferent Mechanisms in Children with Auditory Processing Disorders

**DOI:** 10.3389/fnhum.2014.00860

**Published:** 2014-10-27

**Authors:** Srikanta K. Mishra

**Affiliations:** ^1^Department of Special Education and Communication Disorders, New Mexico State University, Las Cruces, NM, USA

**Keywords:** auditory processing disorders, medial olivocochlear reflex, otoacoustic emissions

## Abstract

Auditory processing disorder (APD) affects about 2–5% of children. However, the nature of this disorder is poorly understood. Children with APD typically have difficulties in complex listening situations. One mechanism thought to aid in listening-in-noise is the medial olivocochlear (MOC) inhibition. The purpose of this review was to critically analyze the published data on MOC inhibition in children with APD to determine whether the MOC efferents are involved in these individuals. The otoacoustic emission (OAE) methods used to assay MOC reflex were examined in the context of the current understanding of OAE generation mechanisms. Relevant literature suggests critical differences in the study population and OAE methods. Variables currently known to influence MOC reflex measurements, for example, middle-ear muscle reflexes or OAE signal-to-noise ratio, were not controlled in most studies. The use of potentially weaker OAE methods and the remarkable heterogeneity across studies does not allow for a definite conclusion whether or not the MOC reflex is altered in children with APD. Further carefully designed studies are needed to confirm the involvement of MOC efferents in APD. Knowledge of efferent functioning in children with APD would be mechanistically and clinically beneficial.

## Introduction

As many as 5% of all children with clinically normal audiograms and no obvious auditory pathology experience listening difficulty in background noise (Chermak and Musiek, 1997; Hind et al., 2011). Other researchers have reported a higher prevalence (20%) of listening problems in children based on data from a large pediatric audiology clinic (Moore and Hunter, 2013). This condition of listening deficits despite a normal audiogram has often received a clinical label called auditory processing disorder (APD).

### Mechanisms of APD

Despite research spanning more than two decades, the term APD is poorly defined and refers to a collection of different functional impairments (Jerger and Musiek, 2000; American Speech-Lanuage-Hearing Association, 2005; American Academy of Audiology, 2010). Children with APD typically have difficulties in complex listening situations, such as understanding speech in background noise or understanding rapid or degraded speech. However, the mechanisms of APD are unclear.

The conventional hypothesis proposes that APD results from impaired “bottom-up” sensory processing and abnormal neural representation of complex acoustic signals, e.g., speech. This may involve lesions in the central auditory nervous system or functional impairment of basic auditory processing (Jerger and Musiek, 2000; Musiek et al., 2005; Richard, 2011); the central auditory system begins in the cochlear nucleus of the medulla and ends with the auditory cortex. However, some researchers argue that the problem may be entirely cognitive, primarily affecting attention, memory or language processing, and exerting a non-specific effect on perception (Moore, 2006, 2012; Moore et al., 2010, 2011), while others claim that APD and attentional deficits are not always related (Sharma et al., 2009). Further, there is a continuing discussion on the relationship between APD and other neurodevelopmental disorders such as specific language impairment and dyslexia (King et al., 2003; Sharma et al., 2006, 2009; Dawes et al., 2008; Ferguson et al., 2011; Moore and Hunter, 2013).

Recently, Ludwig et al. (2014) demonstrated that children with (suspected) APD with normal peripheral function [i.e., normal audiograms, stapedial reflexes, and otoacoustic emissions (OAEs)] but no associated language/reading impairments have, in fact, impaired sensory processing, manifested as a discrimination deficit in spectral and temporal domains. Their results clearly imply that APD, as a distinct clinical entity, cannot be entirely dismissed. They suggested that dichotic listening deficits in these children could be influenced by the subcortical efferent auditory system, specifically the medial olivocochlear (MOC) efferents. Accordingly, it is plausible that processing deficits expressed in the ascending auditory system could reflect the “top-down” influences from cortical centers that may exert their influence on the auditory cortex, and be conveyed to auditory periphery via the efferent pathways (Moore and Hunter, 2013).

Another potential mechanism of speech-in-noise deficits despite a normal audiogram is hypothesized to be reduced temporal encoding precision of supra-threshold sound – termed as cochlear neuropathy (Bharadwaj et al., 2014). This can manifest both behaviorally, for example, frequency modulation discrimination, and in subcortical steady state responses in humans. Whether this is the same as APD is currently not known. Such lack of consensus in APD has affected diagnosis and management of this poorly understood disorder. For instance, one of the major issues in clinical assessment of APD is that most diagnostic tests lack clear scientific underpinnings and empirical evidence.

### Medial olivocochlear inhibition

Regardless of whichever hypothesis of the pathophysiology of APD may turn out to be true, the descending system plays a significant role in hearing and auditory learning (Suga et al., 2000, 2002; Xiao and Suga, 2002; Suga and Ma, 2003; Bajo et al., 2010). The auditory system has an elaborate system of descending, efferent neural pathways that extend to the cochlea (Suga et al., 2000; Xiao and Suga, 2002). Efferent effects shape or modulate the bottom-up afferent processing. This review focuses on the medial efferents (Fex, 1962; Guinan, 2006). Auditory nerve fibers innervate reflex interneurons in the contralateral posteroventral cochlear nucleus. Axons of these interneurons cross the brainstem ventrally and innervate the medial efferent neurons on the ipsilateral side. These neurons project to the ipsilateral cochlea through the uncrossed olivocochlear bundle and modulate outer hair cell (OHC) activity (Guinan, 2006). The OHC functioning can be measured by OAEs. OAEs are sounds of cochlear origin, which can be recorded by a small microphone fitted into the ear canal. They are generated by the motion of the OHCs as they respond to auditory stimulation (Kemp, 1978).

In humans, acoustic stimulation of the MOC system results in altered cochlear amplification that is often described in terms of changes in amplitude and phase of OAEs, and is typically termed OAE suppression or MOC reflex (Guinan, 2006, 2010). In this report, the term MOC reflex is used. Animal work suggest a “MOC unmasking” hypothesis in which stimulation of the MOC efferents reduces cochlear responses to continuous noise, allowing greater responsiveness to transient signals embedded in noise (Winslow and Sachs, 1988; Kawase and Liberman, 1993; Kawase et al., 1993). While a complete understating of the role of the human MOC system in hearing is emerging (Guinan, 2006, 2010), one of the putative functions of this system is to aid in listening-in-noise (Andéol et al., 2011; de Boer et al., 2012; Abdala et al., 2014; Mishra and Lutman, 2014).

Since children with APD clinically present listening deficits in noise, a potential involvement of the MOC efferents has been investigated by some researchers as one of the underlying mechanisms of the APD (Muchnik et al., 2004; Clarke et al., 2006; Sanches and Carvallo, 2006; Garinis et al., 2008; Butler et al., 2011). The benefits of measuring MOC inhibition in children with APD are twofold. It could be useful to better define APD and identify the potential mechanisms of this disorder. Additionally, such work may elucidate the functional significance of MOC efferents in listening in complex environments during the developmental period. The potential role of the MOC system in APD pathophysiology, should it be confirmed, would be of significant clinical interest because current APD clinical test batteries lack mechanism-based physiologic tools that are relatively quick (Emanuel, 2002; Emanuel et al., 2011). In addition, it could offer rehabilitation options for the management of APD or its subgroup through auditory training since MOC reflex strength has been shown to improve with training (Veuillet et al., 2007; de Boer and Thornton, 2008).

### Scope of this review

The objective of this review was to critically evaluate the current evidence to determine whether the MOC system is altered in individuals with APD. The literature was examined in the context of current understanding of the generation mechanism of OAEs (Shera and Guinan, 1999) and recent advancements in human MOC work (Guinan, 2006, 2010). Specifically, the included studies were analyzed for study population characteristics (how the APD was defined) and the rigor of the OAE-based MOC tests, for instance, the type of OAEs used, the quality of OAE measurements in terms of signal-to-noise ratio (SNR), techniques used to control middle-ear muscle reflexes (MEMRs), and method of computation of OAE differences.

Considering the controversies that surround APD, it is important to clarify that the intent of this review was not to precisely define APD or to agree/disagree with the concept of APD *per se*.

## Materials and Methods

The PubMed search engine maintained by the U.S. National Library of Medicine (http://www.ncbi.nlm.nih.gov/pubmed/) was queried for multiple specific search terms. The search period spanned more than three decades from January 1980 to April 2013. No other databases were included. The specific search terms used were *(central) APD* and *MOC reflex*, *OAE suppression*, or *efferent inhibition*. Because of the lack of a precise definition of APD, several comorbid conditions, e.g., dyslexia, learning disability (LD), listening problems, and specific language impairment were also included as search terms; however, studies on speech disorders, for example, selective mutism (Bar-Haim et al., 2004), were excluded. The following inclusion criteria were then applied: English language and human subjects. No age criteria were applied. One article written in non-English language was included as the full text was available in English. Studies were also added manually after reviewing the references of the selected journal papers.

Included studies were reviewed for study population characteristics and the rigor of the OAE-based MOC tests. Specifically, studies were examined for the type of OAEs used to assay MOC reflex, the quality of collected OAE data in terms of SNR, techniques used to control MEMRs and method of computation of OAE differences. In addition, a check was performed as to whether the included studies used state-of-the-art OAE methods, e.g., distortion product otoacoustic emission (DPOAE) fine-structure and components analysis (Abdala et al., 2009; Deeter et al., 2009; Henin et al., 2011), transient-evoked OAE wavelet analysis that retains spectral and temporal emission information (Tognola et al., 1997) or stimulus frequency OAEs (SFOAEs; Backus and Guinan, 2007) and controlled for endogenous variables such as auditory attention (Maison et al., 2001; de Boer and Thornton, 2007).

## Results

Using the aforementioned search criteria, nine articles (eight on children and one on adults) were identified (Veuillet et al., 1999, 2007; Muchnik et al., 2004; Clarke et al., 2006; Sanches and Carvallo, 2006; Burguetti and Carvallo, 2008; Garinis et al., 2008; Yalçinkaya et al., 2010; Butler et al., 2011). Their principal methods and results are summarized in Table 1.

**Table 1:** **Summary of the included studies**.

Study	Clinical population	OAE protocol	CAS	MEMR control	MOC reflex	Conclusions
Veuillet et al. (1999)	Learning-impaired (*n* = 14)	CEOAE; linear; 60-72 dB SPL; unknown SNR; MOC reflex was computed by equivalent attenuation method	30 dB SL BBN	Unknown	Reduced relative to controls; relatively lower OAE levels in the learning-impaired group; right ear was more affected	A deficit in inhibitory MOC mechanisms could reflect a more central dysfunction
Muchnik et al. (2004)	APD/LD (*n* = 15)	TEOAE; non-linear; 74 dB SPL; 3 dB SNR	40 dB SL BBN	Clinical ART	Lower compared with control group; more affected in 8.0–20.48 ms range; no relationship between APD test outcomes and MOC reflex strength; higher OAE levels in APD	Reduced auditory inhibitory function in some children with APD who also had difficulty with hearing in noise
Clarke et al. (2006)	SLI (*n* = 18)	TEOAE; linear; 60 dB SPL; unknown SNR	65 dB SPL BBN	Clinical ART	No group differences; no right vs. left ear differences; MOC reflex in right ear and expressive grammar scores were related	No relationship between MOC activity and language impairment
Sanches and Carvallo (2006)	APD (*n* = 36)	TEOAE; linear 60 dB and non-linear 60–80 dB SPL; unknown SNR	60 dB SPL BBN	Unknown	No group differences but lower prevalence of MOC effect in APD group; no effect of linear vs. non-linear OAE recording method; classifying the APD group based on speech-in-noise scores did not change the study outcomes	Abnormal MOC inhibition was significantly more common in the APD groups than in the control group
Veuillet et al. (2007)	Dyslexics (*n* = 38)	CEOAE; 57–69 dB SPL; 4 dB SNR^68^	30 dB SL BBN	Unknown	Stronger reflex in the right than the left ear in controls, but predominated in the left ear in dyslexics; reduced reflex in the right ear in dyslexics; MOC reflex asymmetry changed following training	Deficits in categorical perception were accompanied by abnormalities in MOC reflex asymmetry in dyslexics
Burguetti and Carvallo (2008)	APD (*n* = 38)	TEOAE; unspecified level; linear; 0–5 dB SNR	60–65 dB SPL BBN	Clinical ART	No significant group differences; tendency for stronger reflex in the control group; right ear advantage in controls, but left ear advantage in the APD group	Lack of clear evidence for a reduced MOC inhibition in APD
Garinis et al. (2008)	LD (*n* = 18)	TEOAE; linear; 60 dB SPL; 3 dB SNR	60 dB SPL BBN	Clinical ART	Relatively lower reflex in the left ear in LD; for the right ear, CAS caused an enhancement in OAE levels for LD but a reduction for controls; lower OAE levels in LD	MOC mechanisms differ in adults with LD compared to those with typical learning abilities; this study included adult participants
Yalçinkaya et al. (2010)	Listening problem (*n* = 12)	TEOAE; non-linear 83 dB SPL; 3 dB SNR	40 dB SL	Clinical ART	Reduced reflex at 1000–2000 Hz band in the study group; lower OAE levels for the right ear in the study group	Lower MOC inhibition may be associated with listening problems
Butler et al. (2011)	APD (*n* = 8)	DPOAE; *f*_2_/*f*_1_ = 1.22 and 1.10; *f*_2_ = 2,3 and 4 kHz; *L*_1_/*L*_2_ = 60/55 dB; SNR > 6 dB	60 dB SPL	ART ≥ 70 dB HL	No group differences in MOC reflex, OAE level, or noise floor	No support for an efferent hypothesis for APD

*APD, auditory processing disorder; ART, acoustic reflex threshold; BBN, broad-band noise; CAS, contralateral acoustic stimulation; CEOAEs, click-evoked otoacoustic emissions; DPOAEs, distortion product otoacoustic emissions; LD, learning disability; SLI, specific language impairment; SNR, signal-to-noise ratio; TEOAE, transient-evoked otoacoustic emissions*.

The number of participants in the experimental groups ranged from 8 to 38, and their auditory processing skills were typically characterized by tests recommended by the ASHA (American Speech-Lanuage-Hearing Association, 2005) or their minor variations. Eight studies used TEOAEs or click-evoked (CE) OAEs while one study used DPOAEs for assaying the MOC inhibition. The SNR of the OAE data across studies was most often 3 dB. The MOC-induced OAE shifts were either computed by raw dB differences or by the equivalent attenuation method (Collet et al., 1992). Six studies used the clinical acoustic reflex procedures, but only three reported the threshold levels (Burguetti and Carvallo, 2008; Garinis et al., 2008; Butler et al., 2011). Due to these differences, a meta-analysis of literature was not feasible.

The mean MOC effect size varied from 0.6 to 2.5 dB across ears, frequencies, time windows, study populations, and studies. The main findings could be categorized based on three metrics: OAE level, MOC reflex magnitude, and ear asymmetry in MOC reflex. Three studies (Veuillet et al., 1999; Garinis et al., 2008; Yalçinkaya et al., 2010) reported lower OAE levels in the study group relative to the control group, while one reported relatively higher OAE levels (Muchnik et al., 2004) and the remaining five (Clarke et al., 2006; Sanches and Carvallo, 2006; Veuillet et al., 2007; Burguetti and Carvallo, 2008; Butler et al., 2011) reported no mean group differences. All but two (Clarke et al., 2006; Butler et al., 2011) studies reported that MOC reflex magnitude in the experimental group was reduced in varying degrees compared to the control group. In three (Veuillet et al., 1999, 2007; Burguetti and Carvallo, 2008) of these seven studies, the MOC reflex was lower in the right ear compared to the left ear for the experimental group in contrast to the right ear advantage in the control group. Interestingly, one study in adults with LD reported a MOC-induced increase in TEOAE level for the right ear (Garinis et al., 2008). Two reports attempted to relate the MOC reflex with performance in the behavioral tests; one study reported that the MOC reflex (magnitude and right/left asymmetry) predicted categorical perception in the APD group (Veuillet et al., 2007), while the other showed no such relationship (Muchnik et al., 2004).

None of the studies implemented state-of-the-art techniques such as DPOAE fine-structure and components unmixing, TEOAE wavelet analysis or SFOAEs to assay MOC reflex. Further attention was not considered in any of the studies.

## Discussion

Despite more than two decades of APD research and two decades of OAE–MOC work, only nine studies, even with a broader inclusion criterion for the study group, probed the potential malfunctioning of the MOC pathway in individuals with APD. Comparison across studies is difficult due to differences in the study populations and their auditory processing characteristics, OAE methods and MEMR tests among the existing clinical trials. More importantly, the existing studies used relatively weak MOC test methods. These studies did not consider crucial measurement confounds necessary for valid and reliable quantification of MOC reflex (discussed later). Therefore, the present review suggests that although a MOC efferent hypothesis for modeling APD is appealing, a definitive conclusion regarding whether or not the MOC system is involved in children with APD cannot be made.

### Middle-ear muscle reflexes

One of the major concerns in OAE-based MOC studies is the possibility that the middle-ear muscle reflex contributes to the MOC reflex (or change in OAE) magnitude by influencing the stimulus and/or emissions as they transmit through the middle-ear (Guinan et al., 2003; Guinan, 2006). Depending on the level, MEMR can be evoked by contralateral broad-band noise (BBN) and/or by OAE-evoking stimulus. Included studies either did not use any MEMR test, did not report (Veuillet et al., 1999, 2007; Sanches and Carvallo, 2006) or used clinical acoustic reflex procedures (Muchnik et al., 2004; Clarke et al., 2006; Burguetti and Carvallo, 2008; Garinis et al., 2008; Yalçinkaya et al., 2010; Butler et al., 2011); see Table 1 under MEMR control. Clinical 226-Hz-probe tone procedures have been shown to yield on average 12–14 dB higher acoustic reflex thresholds, for 1000 and 2000 Hz activators, compared to wideband acoustic reflectance methods (Feeney et al., 2003). It is likely that MOC reflex estimation was compromised due to potential MEMR influences in studies that used high click levels to evoke OAEs (Muchnik et al., 2004; Sanches and Carvallo, 2006; Yalçinkaya et al., 2010) and/or high BBN levels to stimulate the MOC pathway (Muchnik et al., 2004; Yalçinkaya et al., 2010).

### OAE signal-to-noise ratio

The noise floor associated with the OAE recording is an important variable for MOC reflex quantification because shifts in OAE level due to noise can be confused with true physiological inhibition. Likewise, lack of contralateral acoustic stimulation (CAS)-induced OAE shifts could be due to lack of inhibition or to low SNR, making the results difficult to interpret. The magnitude of the MOC effect has been reported to be dependent on the quality or SNR of the OAE data (Goodman et al., 2013). While a high SNR is desirable to detect robust MOC effects, a minimum 6 dB SNR has been shown to produce repeatable results (Francis and Guinan, 2010; Mishra and Lutman, 2013). Unfortunately, other than one study (Butler et al., 2011), most studies had low (3 dB) or unspecified SNR (see Table 1 under OAE protocol). Additionally, these studies did not examine potential SNR differences between the study and control groups.

### OAE recording method

Two studies (Muchnik et al., 2004; Yalçinkaya et al., 2010) used the non-linear CEOAE recording method (Kemp et al., 1986) to assay MOC inhibition. Although the non-linear click method of recording CE/TEOAEs eliminates stimulus ringing artifacts, it cancels the linear portion of the emission, potentially removing inhibition information. In contrast, the linear click method captures the entire emission and retains complete MOC effects.

The DPOAE is a vector combination of at least two components, termed as distortion (generated at the overlap of primary tones *f*_1_ and *f_2_*) and reflection, produced at the *f_dp_* (2*f_1_* − *f_2_*) place (Kim, 1980; Shera and Guinan, 1999; Talmadge et al., 1999; Knight and Kemp, 2000). The interaction of these two components in the ear canal produces oscillations in DPOAE level and phase – fine-structure. The phase interference between distortion and reflection components can confound DPOAE-based measures of the MOC reflex, occasionally creating enhancements (Abdala et al., 2009; Deeter et al., 2009). Enhancements are more common at fine-structure dips or minima where the components are out of phase. The MOC effect differentially influences the two components, with greater changes in the reflection component (Abdala et al., 2009, 2013; Deeter et al., 2009). The effect is not merely reduction in DPOAE level, but shifting of the fine-structure pattern mainly through the reflection component (Henin et al., 2011). Without due consideration of these physiologic mechanisms, the MOC-induced DPOAE changes may be inconclusive. For APD work, only one study used DPOAEs (Butler et al., 2011), but without any consideration to fine-structure or component separation.

### Quantification of the MOC reflex

Conventionally, the MOC reflex has been quantified by computing “raw dB differences” between OAEs with and without CAS. Most APD studies used raw dB metric to show MOC effects (Muchnik et al., 2004; Clarke et al., 2006; Sanches and Carvallo, 2006; Yalçinkaya et al., 2010; Butler et al., 2011). Such computation may not be mathematically accurate, particularly when the OAE levels between the study and control groups are different (Veuillet et al., 1999; Muchnik et al., 2004; Garinis et al., 2008; Yalçinkaya et al., 2010). Recent studies have used a variety of novel methods to compute MOC reflex (Abdala et al., 1999; Backus and Guinan, 2007; Deeter et al., 2009; Henin et al., 2011; Mishra and Lutman, 2013, 2014), for instance, a normalized index that considers the baseline OAE level or a vector index that takes into account the phase information. Three studies have specifically shown that use of normalized index provided different results or study outcomes compared to the raw dB index (Backus and Guinan, 2007; Garinis et al., 2011; Mishra and Lutman, 2014).

### Other measurement-related variables

Other OAE- and CAS-related factors, such as slower OAE probe-drifts (Henin et al., 2011; Goodman et al., 2013) and BBN bandwidths (Maison et al., 2000; Lilaonitkul and Guinan, 2009), respectively, could influence MOC reflex estimates. Lack of these specifications in published reports also makes comparison across studies difficult.

Auditory attention, a subject-related variable, has been shown to influence MOC reflex magnitude (Maison et al., 2001; de Boer and Thornton, 2007). Controlling attention by including task-dependent measures might reduce variability. This is highly relevant for comparing APD and control groups, since a well-designed, population-based study demonstrated auditory inattention as one of the underlying mechanisms of APD (Moore et al., 2010). It is plausible that the effect of auditory attention may be different in children with APD. This prediction, however, requires experimental verification.

### Study population

An obvious challenge is the lack of consensus on what constitutes an APD. Consequently, studies have used a bewildering array of tests to recruit clinical participants that lack clear theoretical import. However, this is a generic APD research problem. Until APD is precisely defined, a working *ad hoc* approach could be to define the experimental group as listeners with impaired speech/listening-in-noise performance, with normal peripheral function and with typical speech-language and learning abilities. It would also be informative to test whether a relationship exists between MOC impairment and the behavioral test outcome in these children (Veuillet et al., 2007).

## Summary

This review suggests that the rigor of MOC reflex measurements in children with APD reported in the available literature is not at par with the recent advancements in OAE-based assays of the MOC reflex. Valid and reliable assessment of the efferent system is lacking in the extant studies.

A related outstanding question is whether MOC efferent mechanisms are important for auditory development. Current understanding of the role of the MOC efferents in auditory perception in children is sparse. Limited data exist showing the relationship between the MOC reflex and speech perception in noise in 10–12-year-old (Kumar and Vanaja, 2004) and 13–17-year-old children (Abdala et al., 2014). It is expected that future studies along these lines will use state-of-the-art OAE–MOC tests that would not only help underpin the mechanisms of APD, but would also enhance the current understanding of the functional significance of the MOC system during development. Ultimately, such work may lead to clinically relevant outcomes.

## Conflict of Interest Statement

The author declares that the research was conducted in the absence of any commercial or financial relationships that could be construed as a potential conflict of interest.

## Abbreviations

APD, auditory processing disorder; ART, acoustic reflex threshold; BBN, broad-band noise; CAS, contralateral acoustic stimulation; CE, click-evoked; DPOAE, distortion product; LD, learning disability; MEMR, middle-ear muscle reflex; MOC, medial olivocochlear; OAE, otoacoustic emission; OHC, outer hair cell; SF, stimulus frequency; SLI, specific language impairment; SNR, signal-to-noise ratio; SPL, sound pressure level; TE, transient-evoked.
